# Arterial Hypertension as a Modulator of Cognitive Response to CPAP Therapy in Moderate-to-Severe Obstructive Sleep Apnea

**DOI:** 10.3390/medicina62010168

**Published:** 2026-01-14

**Authors:** Jelena Šarić Jurić, Mirjana Grebenar Čerkez, Darija Birtić, Kristina Kralik, Stjepan Jurić

**Affiliations:** 1 Faculty of Medicine Osijek, Josip Juraj Strossmayer University of Osijek, 31000 Osijek, Croatia; jelenasaricjuric@gmail.com (J.Š.J.); mirjanagrebenar@gmail.com (M.G.Č.); kinder.darija@gmail.com (D.B.); kkralik@mefos.hr (K.K.); 2 Department of Neurology, University Hospital Centre Osijek, 31000 Osijek, Croatia; 3 Department of Otorhinolaryngology and Head and Neck Surgery, University Hospital Centre Osijek, 31000 Osijek, Croatia

**Keywords:** obstructive sleep apnea, arterial hypertension, continuous positive airway pressure, cognitive function, Montreal Cognitive Assessment, P300 event-related potentials, treatment adherence, nocturnal oxygen saturation

## Abstract

*Background and Objectives*: Cognitive deficits are common in obstructive sleep apnea (OSA), and both intermittent hypoxemia and cardiovascular comorbidity may contribute to poorer outcomes. Arterial hypertension (HTN) has been suggested as a potential modifier of cognitive function in OSA, but findings remain inconsistent. This study examined whether HTN influences baseline cognition or cognitive improvement after continuous positive airway pressure (CPAP) therapy in moderate-to-severe OSA and identified predictors of poorer post-treatment cognitive status. *Materials and Methods*: This prospective study involved 71 adults with newly diagnosed moderate-to-severe OSA (AHI ≥ 15). Participants underwent baseline polysomnography, Montreal Cognitive Assessment (MoCA) testing, and P300 assessments. Cognitive impairment was defined as MoCA < 26 and HTN by antihypertensive therapy, documented diagnosis, or repeatedly elevated blood pressure. All participants initiated auto-adjusting CPAP and were reassessed after three months for adherence, residual respiratory indices, MoCA, and P300 parameters. Multivariate logistic regression and receiver operating characteristic (ROC) analyses were used to identify independent predictors of poorer cognitive outcomes. *Results*: CPAP therapy significantly improved apnea severity, daytime sleepiness, global cognition, and P300 latency, while P300 amplitude did not change significantly. After three months, hypertensive and normotensive patients showed similar MoCA scores, respiratory outcomes, and P300 amplitude; P300 latency remained marginally longer in hypertensive individuals. Across multivariate models, lower mean nocturnal oxygen saturation and reduced CPAP adherence independently predicted poorer cognitive outcome at follow-up. CPAP adherence demonstrated greater discriminative ability than mean nocturnal oxygenation. *Conclusions*: In adequately treated moderate-to-severe OSA, HTN did not significantly affect baseline cognition or short-term cognitive recovery with CPAP. Although P300 latency remained slightly prolonged in hypertensive individuals, this difference was marginal and not accompanied by cognitive deficits. Nocturnal oxygenation and CPAP adherence emerged as the strongest predictors of post-treatment cognitive status, underscoring the importance of sustained and effective therapy. These findings suggest that effective CPAP adherence and improved nocturnal oxygenation are crucial for cognitive recovery in OSA patients, regardless of hypertensive status.

## 1. Introduction

### 1.1. Obstructive Sleep Apnea and Cognitive Dysfunction

Obstructive sleep apnea (OSA) is a highly prevalent sleep-related breathing disorder and an important global health issue due to its broad systemic and neurocognitive consequences. It is characterized by recurrent episodes of partial or complete upper airway obstruction during sleep, leading to reduced or absent airflow despite preserved respiratory effort [1]. These respiratory disturbances result in intermittent hypoxemia, repeated arousals, and significant fragmentation of normal sleep architecture, ultimately impairing restorative sleep processes.

Recent epidemiological analyses suggest that OSA is highly prevalent, affecting nearly one billion adults globally. Using an AHI threshold of ≥5 events/h and the 2012 AASM scoring criteria, approximately 425 million adults aged 30–69 years meet criteria for moderate-to-severe disease (AHI ≥ 15 events/h) [2]. Beyond its well-recognized cardiometabolic and cerebrovascular implications, OSA is strongly associated with cognitive dysfunction [3]. Affected individuals frequently demonstrate impairments in attention, executive functioning, memory, vigilance, and visuospatial abilities [4,5,6]. Although the reported prevalence and severity of cognitive deficits vary substantially across studies—largely due to methodological differences—intermittent hypoxemia and sleep fragmentation are consistently identified as central mechanisms contributing to neural injury and cognitive decline.

### 1.2. Assessment of Cognitive Impairment in OSA

Cognitive function in patients with OSA can be assessed through standardized neuropsychological tests as well as electrophysiological measures such as cognitive event-related potentials (ERPs). Among available cognitive screening tools, the Montreal Cognitive Assessment (MoCA) is particularly suitable for use in OSA populations because it provides higher sensitivity and specificity for detecting mild cognitive impairment (MCI) compared with the Mini-Mental State Examination (MMSE). Previous studies have shown that MoCA correctly identifies approximately 72–81% of OSA patients with MCI, with specificity ranging from 72% to 86%, whereas the MMSE demonstrates limited sensitivity and poor diagnostic accuracy in this context [7]. For these reasons, MoCA is considered a more appropriate instrument for capturing the subtle cognitive deficits commonly observed in OSA.

### 1.3. Arterial Hypertension as a Potential Modifier of Cognitive Outcome

Arterial hypertension (HTN) is now recognized as a major risk factor for cognitive decline, Alzheimer’s disease, and vascular dementia [8,9,10]. Longstanding elevations in blood pressure contribute to small vessel disease, impaired cerebral autoregulation, endothelial dysfunction, and reduced β-amyloid clearance, all of which predispose to cognitive impairment [10]. Importantly, HTN and OSA frequently coexist, share overlapping pathophysiological pathways, and may have additive effects on cerebrovascular and cognitive function [11]. Although both conditions independently increase the risk of cognitive decline, it remains unclear whether HTN further modifies or exacerbates the cognitive consequences of OSA.

### 1.4. Cognitive Effects of CPAP Therapy and Existing Knowledge Gaps

Continuous positive airway pressure (CPAP) is the first-line treatment for OSA and effectively improves sleep architecture, reduces the apnea–hypopnea index, and alleviates daytime symptoms [12]. Evidence supporting cognitive recovery after CPAP is growing, although findings remain heterogeneous [4,13,14,15]. Despite growing evidence for cognitive benefits of CPAP, it remains unclear whether all patients experience comparable recovery. HTN, a common and biologically relevant comorbidity, could theoretically limit or modify the extent of cognitive improvement due to its chronic vascular effects. However, the extent to which HTN influences neurocognitive response to CPAP therapy has not been systematically investigated. Addressing this question has important clinical implications, given the high prevalence of both conditions and the potential for early intervention to prevent long-term cognitive decline.

### 1.5. Study Rationale and Objectives

Therefore, the aim of this study was to determine whether HTN influences baseline cognitive function and short-term cognitive recovery—assessed using both the MoCA and P300 event-related potentials—after three months of CPAP therapy in patients with moderate-to-severe OSA. Specifically, we compared MoCA score and P300 parameters between hypertensive and normotensive individuals before and after treatment and explored predictors of poorer post-treatment cognitive outcomes.

## 2. Materials and Methods

This prospective observational study was conducted at the Department of Neurology, University Hospital Centre Osijek, between September 2019 and May 2025. A total of seventy-one adults aged 18–65 years with newly diagnosed moderate-to-severe OSA were enrolled. All participants received a detailed explanation of the study protocol and provided written informed consent prior to inclusion.

### 2.1. Inclusion and Exclusion Criteria

Eligible individuals were men and women between 18 and 65 years of age with moderate-to-severe OSA confirmed on overnight polysomnography, defined by an apnea–hypopnea index (AHI) of ≥15 events per hour. Eligibility was limited to patients with no previous use of CPAP or other therapeutic interventions for OSA, who were capable of completing cognitive testing and exhibited normal findings on baseline neurological and general medical evaluations, as documented in the clinical file.

**Figure 1:**
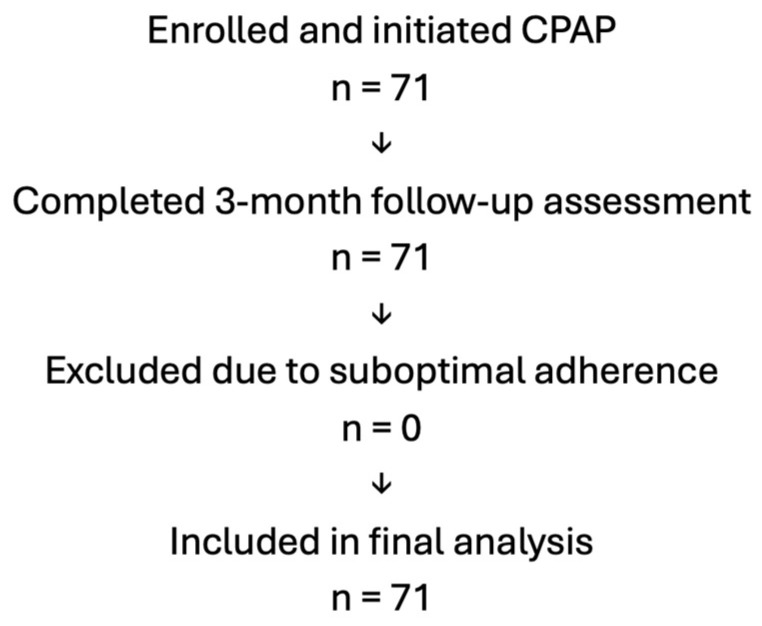
Participant flow diagram. Information on the total number of patients screened and excluded prior to enrollment was not systematically recorded, as this study was conducted within routine clinical practice. All enrolled participants completed follow-up and met the predefined CPAP adherence threshold; therefore, no exclusions were required on this basis.

Exclusion criteria encompassed any prior therapeutic intervention for OSA, the presence of mild OSA, primary central sleep apnea, or other concomitant sleep disorders. Individuals with chronic pulmonary disease, ongoing alcohol or substance misuse, or inability or unwillingness to initiate CPAP therapy were not eligible for inclusion. CPAP adherence was evaluated at the three-month follow-up, and all participants who completed follow-up satisfied the predefined adherence threshold (≥4 h per night on ≥70% of monitored nights), so no exclusions were required on this basis. Additional exclusion criteria comprised medical, neurological, or psychiatric conditions with established effects on cognitive performance, including complicated diabetes mellitus, a history of cerebrovascular events, major depressive disorder, neurodegenerative disorders, and the use of psychoactive medications. Participant flow is illustrated in Figure 1. Detailed records of patients screened and excluded prior to enrollment were not systematically retained, as the study was conducted in routine clinical practice.

### 2.2. Assessment of HTN

HTN was not an inclusion or exclusion criterion. HTN was defined using several possible indicators. Participants were classified as hypertensive if they were already receiving at least one antihypertensive medication, if their average blood pressure (BP) exceeded 140/90 mm Hg based on 10 consecutive measurements collected over 5 days, or if their medical history documented a prior diagnosis of HTN. Participants were classified as hypertensive or normotensive for the purpose of subgroup comparisons and multivariate analyses evaluating the impact of HTN on cognitive performance and treatment response.

### 2.3. Baseline Assessments

All participants underwent a structured neurological examination and anthropometric evaluation including weight, height, and body mass index (BMI). Subjective sleepiness and OSA risk were assessed using the Epworth Sleepiness Scale (ESS), STOP-Bang questionnaire, and Berlin questionnaire. Cognitive performance was evaluated using the MoCA, selected due to its superior sensitivity and specificity for detecting OSA-related mild cognitive impairment compared with the Mini-Mental State Examination [6]. Cognitive status was classified based on the MoCA, with scores <26 indicating impaired cognitive function and scores ≥26 indicating preserved cognitive performance [16]. Hearing thresholds were verified as normal using pure-tone audiometry to ensure reliable acquisition of P300 ERPs.

### 2.4. Polysomnography

OSA was diagnosed by overnight attended polysomnography (PSG) performed with the Alice 6 Diagnostic Sleep System (Philips Respironics, Murrysville, PA, USA), and recordings were analyzed with Sleepware G3 software (version 3.9.1). The PSG montage included a 16-channel EEG according to the international 10–20 system, bilateral electrooculography, chin and tibialis electromyography, lead II electrocardiography, thoracoabdominal respiratory effort belts, nasal and oral airflow sensors, and pulse oximetry. Sleep staging and respiratory scoring were performed according to the American Academy of Sleep Medicine (AASM) 3.0 criteria.

Recorded variables included total sleep time, sleep efficiency, sleep stage distribution, sleep onset and REM latencies, wake after sleep onset, and the number of sleep cycles. Respiratory parameters included total AHI, REM-AHI, NREM-AHI, and detailed indices of obstructive, central, and mixed apneas and hypopneas. Measures of nocturnal oxygenation, including the oxygen desaturation index, mean oxygen saturation, and minimum SpO_2_, were obtained. Mean nocturnal oxygen saturation (mean SpO_2_) was defined as the average oxygen saturation during total sleep time. Minimum SpO_2_ represented the lowest oxygen saturation recorded during sleep. The oxygen desaturation index (ODI) was defined as the number of ≥3% desaturation events per hour of sleep. The percentage of total sleep time spent with oxygen saturation below 90% (T90) was not available for all participants and was therefore not included in the statistical analyses. Snoring metrics, cardiovascular parameters, and limb movement indices were also recorded. The full PSG profile for this cohort is provided in the corresponding tables of the dataset.

### 2.5. CPAP Therapy and Adherence

All patients who met clinical and polysomnographic criteria were enrolled regardless of anticipated CPAP adherence. Objective CPAP adherence was assessed at the three-month follow-up using device-recorded data. CPAP adherence was defined according to the Centers for Medicare and Medicaid Services (CMS) criterion—use of the device for ≥4 h per night on ≥70% of monitored nights [17]. Only participants who met this predefined adherence threshold contributed data to the final analytical dataset. Recorded parameters included average nightly usage, percentage of nights meeting the adherence threshold, residual AHI, and therapeutic pressure settings.

### 2.6. Event-Related Potentials

Cognitive ERPs were recorded using a Medelec Synergy EMG/EP system (VIASYS Healthcare Inc., Madison, WI, USA) employing a standard auditory oddball paradigm. Frequent standard tones (1000 Hz) and infrequent target tones (2000 Hz) were presented binaurally at 80 dB SPL with a duration of 50 ms. EEG activity was recorded from Fz, Cz, and Pz electrodes, referenced to linked mastoids, with impedances maintained below 5 kΩ. Signals were band-pass filtered between 0.1 and 70 Hz, with a 50 Hz notch filter applied. Epochs spanning 0–700 ms post-stimulus were analyzed. P300 latency and amplitude served as the principal electrophysiological markers of cognitive processing.

### 2.7. Additional Measurements

Audiometry was performed in a sound-attenuated booth using a GSI AudioStar Pro audiometer to exclude clinically significant hearing impairment. Blood pressure was measured prior to PSG with a Ri-san^®^ manual sphygmomanometer (Rudolf Riester GmbH, Jungingen, Germany). Fasting capillary glucose was assessed the following morning with an Accu-Chek Performa glucometer (Roche Diagnostics, Basel, Switzerland).

### 2.8. Follow-Up

After three months of CPAP treatment, participants returned for reassessment. The ESS, MoCA, and P300 ERP evaluations were repeated, and CPAP adherence data, residual respiratory indices, and device-derived parameters were re-examined. All eligible patients were enrolled irrespective of anticipated CPAP adherence. CPAP adherence was assessed objectively at follow-up, and exclusion due to suboptimal adherence occurred after follow-up when device usage data became available. Antihypertensive therapy remained stable throughout the three-month follow-up. No medication changes or additions were made during this period.

### 2.9. Statistical Analysis

Categorical variables were expressed as absolute and relative frequencies. Differences between categorical variables were analyzed using Chi-square test and Fisher’s exact test. The Shapiro–Wilk test assessed normality of continuous variables. Continuous data were summarized as medians with interquartile ranges. Within-group comparisons were performed using the Wilcoxon signed-rank test, while differences between groups were analyzed using the Mann–Whitney U test with Hodges–Lehmann median difference and 95% confidence interval. Independent predictors of cognitive outcomes after three months of CPAP therapy were identified using stepwise multivariate logistic regression. Receiver operating characteristic (ROC) curve analysis was used to determine optimal cut-off values for significant predictors. All *p* values were two-tailed, and the significance level was set at α = 0.05. Statistical analyses were conducted using MedCalc^®^ Statistical Software version 23.3.7 (MedCalc Software Ltd., Ostend, Belgium; 2025) and IBM SPSS Statistics v23 (IBM Corp., Armonk, NY, USA).

## 3. Results

The study included 71 participants, of whom 57 were men (80.3%) and 14 were women (19.7%). HTN was present in 41 participants (57.7%). Gender distribution according to HTN status was without significant difference between groups. Baseline demographic, anthropometric and polysomnographic characteristics are presented in Table 1 and Table 2.

Baseline (before CPAP) characteristics stratified by HTN status are presented in Table 2. Hypertensive participants were significantly older and exhibited longer REM latency, greater wake after sleep onset, fewer sleep cycles, and lower sleep efficiency compared with normotensive participants. Other anthropometric, respiratory, cognitive, and electrophysiological baseline parameters, including OSA severity indices, oxygenation measures, baseline MoCA scores, and P300 characteristics, did not differ significantly between groups (Table 2).

CPAP therapy produced significant improvements across respiratory, subjective sleepiness, cognitive, and electrophysiological measures. AHI decreased from a median of 56.2 events/h to 3.0 events/h (difference −53.4; *p* < 0.001), and daytime sleepiness (ESS) improved from 11 to 2 (*p* < 0.001).

**Figure 2:**
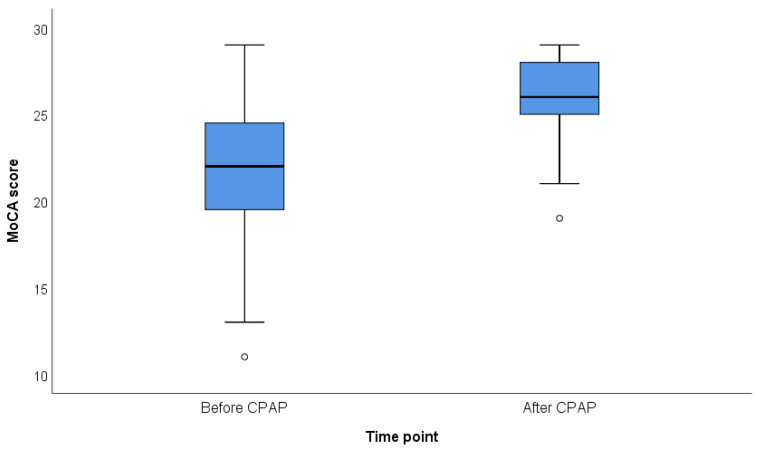
Distribution of MoCA scores before and after CPAP therapy. (Box-and-whisker plots illustrate the distribution of Montreal Cognitive Assessment (MoCA) scores at baseline (before CPAP) and after three months of CPAP therapy. The central line represents the median, boxes indicate the interquartile range (IQR), and whiskers denote minimum and maximum values. Individual outliers are shown as points).

Cognitive performance improved significantly following CPAP treatment. The total MoCA score increased by a median of 4 points (from 22 to 26; *p* < 0.001) after three months of CPAP therapy. Event-related potential measures demonstrated reductions in P1 and P300 latencies (−5 ms and −23 ms, respectively; *p* = 0.02 and *p* < 0.001). P300 amplitude showed a non-significant increase (Table 3 and Figure 2).

After three months of CPAP therapy, respiratory outcomes, ESS scores, and MoCA score did not differ significantly between hypertensive and normotensive participants P300 latency was slightly longer in hypertensive individuals (median 319 ms vs. 307 ms) with borderline statistical significance (*p* = 0.05). No other ERP components differed between groups (Table 4).

At both baseline and follow-up, the prevalence of impaired cognitive function did not differ significantly between participants with and without HTN (Table 5).

A multivariate logistic regression analysis was performed to identify independent predictors of poorer cognitive status at follow-up. The following variables were entered into the model: age, BMI, WASO, ODI, minimum oxygen saturation, mean oxygen saturation, percentage of CPAP use, average nightly CPAP duration, ESS score before and after treatment, and AHI before and after CPAP therapy. The analysis was conducted both without and with adjustment for the presence of HTN.

Without adjustment for HTN, the overall model was statistically significant (χ^2^ = 10.18, *p* = 0.006), explaining 14% of the variance according to Cox & Snell and 19% according to Nagelkerke R^2^, with a correct classification rate of 70%.

After adjustment for HTN, the model remained statistically significant (χ^2^ = 10.4, *p* = 0.02), explaining the same proportion of variance (14 to 19%) and correctly classifying 71% of cases. Importantly, the predictive contributions of mean oxygen saturation and the proportion of CPAP use remained unchanged after controlling for HTN, indicating that HTN does not attenuate their independent association with poorer cognitive outcome. A higher percentage of CPAP use was associated with increased odds of being classified into the poorer cognitive outcome group in the logistic regression models, both before and after adjustment for HTN (Table 6).

**Figure 3:**
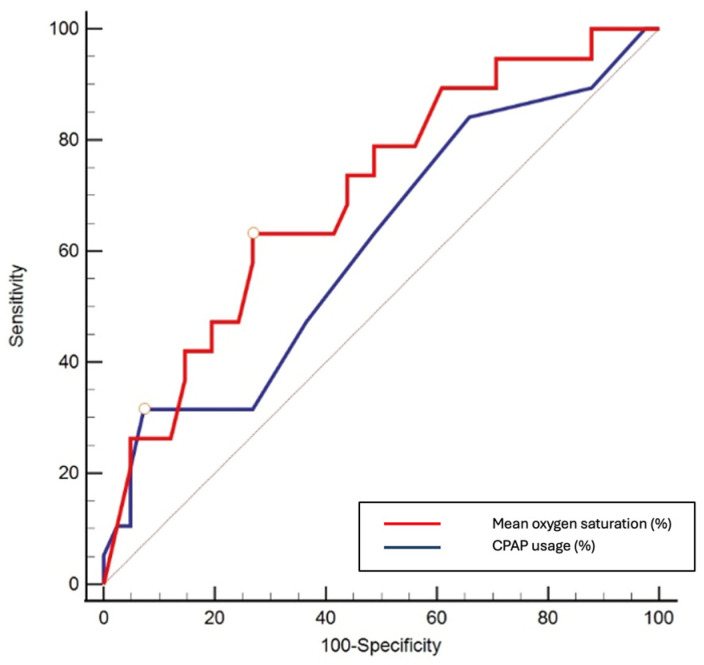
ROC curve comparing mean nocturnal oxygen saturation and CPAP adherence in distinguishing participants with good versus poorer cognitive status according to MoCA scores after therapy.

Consistent with the logistic regression findings, a higher proportion of CPAP use demonstrated a slightly better ability to classify participants with poorer cognitive outcome compared with mean nocturnal oxygen saturation, as reflected by a higher AUC (0.645 vs. 0.630) and a greater Youden index. Although neither parameter showed strong discriminative accuracy, CPAP adherence exhibited high sensitivity (96%) for identifying individuals classified as having poorer cognitive performance. These findings should be interpreted as reflecting classification performance rather than a causal effect of CPAP use on cognitive outcome (Table 7 and Figure 3).

## 4. Discussion

This study aimed to determine whether HTN influences baseline cognitive function or cognitive recovery after three months of CPAP therapy in patients with moderate-to-severe OSA. The results show that HTN does not meaningfully affect MoCA-defined cognitive impairment: both hypertensive and normotensive patients exhibited similar improvements in total MoCA scores and P300 amplitudes, and HTN did not alter the predictive value of mean nocturnal oxygen saturation or CPAP adherence for cognitive outcome. Although P300 latency remained slightly prolonged in hypertensive individuals, this difference reached only borderline statistical significance and was not accompanied by differences in other ERP components or MoCA performance, suggesting limited clinical relevance. These findings run counter to prevailing hypotheses suggesting that HTN may potentiate cognitive vulnerability in OSA and attenuate treatment-related cognitive gains, indicating instead that short-term cognitive improvement with CPAP appears robust across vascular risk profiles [17,18].

Three months of CPAP therapy resulted in clinically relevant improvements across respiratory indices, subjective sleepiness, cognitive function, and electrophysiological markers of cognition. The substantial reduction in AHI confirms effective stabilization of breathing during sleep and aligns with established evidence demonstrating the efficacy of CPAP in moderate to severe OSA [18]. Corresponding improvements in daytime sleepiness further illustrate the functional benefits of consistent treatment. Cognitive outcomes showed notable enhancement following therapy. Although median MoCA scores increased from 22 to 26, indicating meaningful recovery of cognitive functioning, these gains should not be interpreted as complete restoration. A subset of participants continued to demonstrate measurable deficits at follow-up, which is consistent with the chronic nature of OSA-related neural alterations and the relatively short duration of treatment in the present study. Electrophysiological findings were in line with behavioral data. Shorter P300 latencies suggest more efficient attentional processing following treatment. The amplitude findings showed a similar direction of change: although P300 amplitude increased after three months of CPAP therapy, the change was not statistically significant. This is consistent with previous studies reporting that CPAP tends to produce clearer and earlier improvements in P300 latency than in amplitude, particularly in middle-aged and older adults [19,20,21,22]. Given that P300 amplitude is known to decline with age, age-related variability within our cohort may have reduced the likelihood of detecting a significant treatment effect [23]. Thus, while latency reductions reflect a robust electrophysiological response to therapy, amplitude recovery may be more gradual or more sensitive to age-dependent influences.

Several physiological mechanisms may explain these cognitive and electrophysiological changes. Improved nocturnal oxygenation is expected to reduce oxidative stress, mitochondrial dysfunction, and neuroinflammation—processes implicated in functional disruption of the hippocampal, parietal, and prefrontal regions [24,25]. Mitigation of intermittent hypoxemia likely supports synaptic plasticity and re-establishes more efficient neural signaling within executive and attentional pathways. These processes, collectively, may account for the pattern of cognitive recovery observed in this cohort.

HTN is widely recognized as a risk factor for cognitive decline, with numerous epidemiologic studies demonstrating its association with reduced white matter integrity, impaired executive function, and slowed information processing [26]. Structural and functional brain alterations linked to long-standing HTN—including microvascular remodeling, impaired cerebral autoregulation, and increased white matter hyperintensities—might therefore be expected to influence cognitive or electrophysiological responses to CPAP therapy [27,28]. However, in our cohort, hypertensive and normotensive participants showed comparable improvements in respiratory outcomes, subjective sleepiness, and cognitive performance after three months of CPAP treatment. This suggests that short-term CPAP efficacy is not substantially moderated by vascular comorbidity. The absence of baseline differences in OSA severity, oxygenation, and cognitive measures strengthens the interpretation that HTN did not independently modify short-term cognitive response to CPAP. The only parameter that approached significance was P300 latency, which remained slightly prolonged in hypertensive individuals. This subtle latency difference is consistent with prior evidence showing that HTN disproportionately affects processing speed and frontoparietal network efficiency, while sparing amplitude-related markers of attentional resource allocation [29]. Our findings are broadly consistent with previous work demonstrating that HTN contributes to cognitive vulnerability in patients with OSA [30], although, in our cohort, HTN did not substantially modify the cognitive or respiratory response to CPAP. Large clinical datasets, such as RIFADE, have shown that untreated HTN can attenuate or even negate the cognitive benefits of OSA treatment, whereas combined management of both conditions yields the most favorable cognitive trajectories [31]. Similarly, a recent study in hypertensive adults demonstrated that those with coexisting OSA exhibited significantly poorer MoCA and MMSE performance and a markedly higher prevalence of cognitive impairment compared with hypertensive individuals without OSA, with OSA emerging as an independent predictor of cognitive dysfunction [32]. These observations support the notion that HTN and OSA exert additive neurocognitive burden, but they also underline that effective treatment—particularly CPAP—can mitigate this risk. The absence of group differences in our study may therefore reflect adequate BP control, effective OSA treatment, and the relatively short disease duration in our sample.

To better understand which factors contribute to variability in cognitive recovery, we performed a multivariate logistic regression analysis including a broad range of demographic, sleep-related, and treatment-related variables. Because arterial hypertension (HTN) has been implicated as a potential modifier of cognitive vulnerability, analyses were conducted both with and without adjustment for HTN. Across both models, mean nocturnal oxygen saturation and the proportion of CPAP use remained the only significant independent predictors of classification into poorer cognitive status at follow-up, while the presence of HTN did not alter their predictive strength or direction.

The observed association between greater CPAP adherence and poorer cognitive outcome may appear counterintuitive. This finding is most plausibly explained by confounding by indication, whereby individuals with more severe baseline hypoxemia or greater cognitive vulnerability are more likely to adhere closely to CPAP therapy. Accordingly, CPAP adherence in the present analysis should be understood as a short-term marker of baseline disease burden influencing outcome classification, rather than as a measure of treatment efficacy over time.

In addition, the three-month follow-up period may be insufficient to capture the full trajectory of cognitive recovery in patients with more advanced baseline impairment. Individuals with higher disease burden may therefore demonstrate excellent adherence yet fail to achieve satisfactory cognitive recovery at early follow-up, whereas longer treatment durations (e.g., 6–12 months) may be required to observe more substantial cognitive gains. Consistent with this interpretation, longitudinal evidence indicates that meaningful cognitive benefits of CPAP therapy emerge with sustained treatment exposure. For example, Velescu et al. reported that patients adhering to CPAP over one year experienced significant improvements in global cognition—particularly in attention and delayed recall—whereas non-adherent patients showed no comparable gains [33]. These data emphasize that adherence is not merely a facilitator but a central determinant of CPAP-related cognitive improvement, reinforcing the notion that consistent device use is essential for achieving meaningful neurocognitive recovery in moderate-to-severe OSA. It is important to emphasize that the observed association between higher CPAP usage and poorer cognitive classification at follow-up should not be interpreted as evidence of a detrimental effect of CPAP on cognition. Given the short follow-up period and the exclusion of non-adherent users from the analytic dataset, this counterintuitive pattern likely reflects clinical or behavioral confounding rather than treatment-related harm. Individuals with more severe baseline hypoxemia or greater perceived cognitive vulnerability may have been more motivated to adhere more strictly to therapy, which could temporarily bias short-term outcome classification. Additionally, ceiling and practice effects in the MoCA may limit sensitivity to detect early cognitive recovery in the subgroup with worse baseline function. The classification model used in this study should therefore be interpreted as exploratory rather than causal, and future studies with larger sample sizes and longer treatment duration will be better positioned to perform sensitivity analyses incorporating baseline cognition and alternative cognitive outcome definitions.

Taken together, these findings indicate that treatment-related factors—particularly consistent CPAP use and adequate nocturnal oxygenation—play a more prominent role in shaping post-treatment cognitive outcomes than HTN itself. ROC analysis further demonstrated that CPAP adherence provided greater sensitivity for identifying individuals at potentially increased risk of suboptimal cognitive recovery than mean nocturnal oxygen saturation, although overall discriminative accuracy was modest. From a clinical perspective, these results highlight that, among treatment-related factors, CPAP adherence and nocturnal oxygenation represent the most robust predictors of cognitive improvement, underscoring the importance of patient engagement and optimal therapy compliance for neurocognitive recovery in OSA.

This study has several limitations. The sample size was relatively modest, which may have limited the ability to detect subtle effects, particularly interactions between HTN and cognitive response to CPAP therapy.

Although the use of the MoCA and P300 event-related potentials enabled a combined behavioral and electrophysiological assessment of cognition, the cognitive evaluation remains relatively limited. MoCA is a global screening instrument and does not allow for detailed domain-specific analyses, particularly of executive functions and processing speed. Given that HTN may preferentially affect these cognitive domains through cerebrovascular mechanisms, subtle group differences may therefore have gone undetected. An additional limitation is the potential influence of practice effects on repeated MoCA administration. As the same MoCA version was used at baseline and follow-up, some degree of score improvement may reflect retest learning rather than true cognitive change. Therefore, observed improvements in MoCA scores should be interpreted cautiously and may represent an overestimation of treatment-related cognitive gains.

In addition, HTN was defined based on prior diagnosis and/or antihypertensive treatment, without detailed information on disease duration, severity, or longitudinal blood pressure control during follow-up. Long-standing or poorly controlled HTN may exert stronger cerebrovascular effects and could influence cognitive outcomes independently of sleep-disordered breathing and CPAP treatment. The lack of these data limits more refined interpretation of HTN-related differences in cognitive response to therapy.

Finally, the three-month follow-up primarily captures early treatment effects and does not allow evaluation of longer-term cognitive trajectories or the potential modifying influence of HTN duration and long-term blood pressure control.

Future research should include larger, well-characterized cohorts, longer follow-up periods, and more comprehensive neuropsychological testing to better characterize cognitive changes associated with OSA and its treatment. In addition, studies addressing CPAP adherence as a stratification variable rather than an exclusion criterion, together with detailed characterization of vascular comorbidities—particularly the duration and degree of control of HTN—are needed to clarify their combined impact on neuropsychological performance and event-related potential components.

Taken together, these findings suggest that, when adequately controlled, HTN does not exert a measurable influence on baseline cognitive status or short-term cognitive improvement with CPAP in moderate-to-severe OSA. Although hypertensive participants demonstrated mildly prolonged P300 latencies, this borderline difference was not accompanied by deficits in MoCA performance or other ERP components, underscoring its limited clinical relevance. Instead, treatment-related factors—particularly consistent CPAP use and adequate nocturnal oxygenation—emerged as the key determinants of cognitive outcome. These results reinforce the importance of optimizing adherence and respiratory stability to support early neurocognitive recovery in OSA, while indicating that well-managed HTN is unlikely to attenuate short-term cognitive benefits of CPAP therapy.

## 5. Conclusions

In this prospective study of patients with moderate-to-severe OSA, HTN did not exert a measurable influence on baseline cognitive performance or short-term cognitive recovery after three months of CPAP therapy. Improvements in MoCA scores and P300 latencies were comparable between hypertensive and normotensive participants, and HTN did not alter the predictive value of mean nocturnal oxygen saturation or CPAP adherence for cognitive outcomes. Instead, treatment-related factors—particularly consistent nightly CPAP use and adequate nocturnal oxygenation—emerged as the primary determinants of cognitive status at follow-up. Although early cognitive gains were evident, residual deficits persisted in a subset of patients, underscoring the need for continued monitoring and longer-term evaluation. Overall, these findings highlight the critical importance of optimizing CPAP adherence and reducing OSA-related physiological stressors to support cognitive recovery, while indicating that well-controlled HTN may not substantially modify the early cognitive response to therapy.

## Figures and Tables

**Table 1:** Distribution of participants by gender and HTN status.

	Number (%) of Participants			*p* * Value
	Without HTN (n = 30)	With HTN (n = 41)	Total (n = 71)	
Gender				
M	27 (90)	30 (73)	57 (80)	0.08
F	3 (10)	11 (27)	14 (20)	

* Chi-square test. Abbreviations: HTN—HTN.

**Table 2:** Baseline (pre-CPAP) characteristics of participants stratified by HTN status.

	Median (IQR)			^†^ Difference (95% CI)	*p* * Value
	Total (n = 71)	Without HTN (n = 30)	With HTN (n = 41)		
Age (years)	51 (44–58)	46 (37.5–51.25)	56 (49.5–59.5)	10 (5 to 14)	**<0.001**
Body weight (kg)	106 (92–136)	103.5 (91.5–130)	111 (92.5–138.5)	4.5 (−6 to 18)	0.34
Body height (cm)	176 (170–183)	177.5 (171.5–183.5)	174 (168–181)	−4 (−8 to 1)	0.09
BMI (kg/m^2^)	34.3 (30.8–42.8)	32.95 (29.08–37.69)	35.9 (30.85–45.25)	2.9 (−0.2 to 6.5)	0.07
Systolic blood pressure (SBP) (mmHg)	130 (125–135)	127.5 (120–135)	130 (125–135)	5 (0 to 10)	0.18
Diastolic blood pressure (DBP) (mmHg)	80 (75–80)	77.5 (75–80)	80 (75–85)	0 (0 to 5)	0.09
Sleep architecture					
Total recording time (min)	465.8(447–502.2)	460.1 (445.9–512.2)	467.8 (443.2–492.9)	−5.7 (−29.5 to 15.7)	0.57
Total sleep time (min)	403 (371.5–448)	422 (384.3–471.6)	402 (358–441.5)	−26 (−54.5 to 7)	0.13
NREM Stage 1 (%)	10.9 (7.9–15.3)	10 (7.3–13.4)	13 (9.3–16.3)	2.7 (0.3 to 5.4)	**0.03**
NREM Stage 2 (%)	58.3 (52–65.1)	55.35 (51–62.7)	59.1 (53.6–65.1)	2.8 (−3.1 to 7.2)	0.29
NREM Stage 3 (%)	17.5 (12.8–25.5)	19.65 (12.8–27.0)	16.8 (13.1–20.9)	−2.9 (−7.6 to 1.3)	0.18
REM sleep (%)	10.7 (8.4–15.9)	12.25 (9.5–15.4)	9.5 (7.3–16.1)	−2.1 (−4.4 to 0.4)	0.10
NREM sleep (%)	89.1 (83.8–91.4)	87.75 (83.8–90.9)	90.3 (83.8–92.8)	2 (−0.7 to 4.8)	0.17
Sleep onset latency (min)	11.5 (6–24.5)	9.15 (5.7–21.3)	12.3 (5.9–27)	1.5 (−2.6 to 7)	0.47
REM latency (min)	124 (101.5–177)	112.5 (87.5–142.4)	143.5 (105.3–213.5)	27.6 (3.5 to 57.5)	**0.03**
WASO (min)	27.9 (8.3–62.1)	10.3 (4.1–28.78)	46.5 (21.1–74.8)	26.1 (11.3 to 41.5)	**<0.001**
Number of sleep cycles	3 (3–4)	4 (3–4)	3 (3–4)	−1 (−1 to 0)	**0.008**
Sleep efficiency (%)	86.4 (81.9–92.8)	91 (85.5–96.2)	84.2 (79.6–88.9)	−6.9 (−10.4 to −3.5)	**<0.001**
Respiratory parameters					
REM AHI (events/h)	62.4 (49.5–75.9)	61 (42–76)	62.4 (51.05–76.4)	3.2 (−7.2 to 14.4)	0.56
NREM AHI (events/h)	59.2 (37.8–72.9)	64.45 (45.93–74.35)	54.8 (31.45–72.65)	−6.5 (−16.2 to 3.3)	0.20
CPAP device parameters					
Average CPAP use (h:min)	5:40 (4:49–6:16)	5:37 (4:49–6:25)	5:41 (4:45–6:16)	0:05 (−0:01 to 0:02)	0.93
CPAP usage (%)	92 (85–99)	93 (86.75–98.93)	90 (78–97.05)	−1.6 (−6 to 1.5)	0.27
AHI (events/h) (before CPAP)	56.2 (40.3–71.7)	56.45 (44.08–74.48)	56.1 (36.25–71.45)	−3.4 (−13.3 to 6.3)	0.45
ESS (daytime sleepiness) (before CPAP)	11 (7–15)	12.5 (10.5–15.25)	10 (6.5–15)	−2 (−4 to 1)	0.17
Total MoCA score (before CPAP)	22 (19–25)	23.5 (19–25.25)	22 (19.5–24)	−1 (−2 to 1)	0.39
P300–Event-related potentials (before CPAP)					
P1 latency (ms)	38 (26–48)	34.5 (24.75–48.5)	41 (26.5–47.5)	3 (−3 to 10)	0.30
N1 latency (ms)	95 (90–108)	99 (90–108)	94 (89.5–108.5)	−2 (−9 to 6)	0.64
P2 latency (ms)	184 (171–198)	185 (172.25–200.25)	181 (170.5–195)	−3.5 (−12 to 8)	0.52
N2 latency (ms)	238 (225–265)	234.5 (223.5–276.5)	240 (228.5–263)	2 (−13 to 14)	0.82
P300 latency (ms)	341 (324–352)	344 (318–360.75)	339 (329.5–349.5)	−1 (−13 to 12)	0.85
P300 amplitude (µV)	9.7 (6–13.9)	10.3 (5.48–14.1)	9.7 (6.25–13.4)	−0.3 (−3.1 to 2.1)	0.77

* Mann–Whitney U test; ^†^ Hodges–Lehmann median difference. Bold value denotes statistical significance. Abbreviations: IQR—Interquartile Range; HTN—Arterial hypertension; NREM—non-rapid eye movement; REM—rapid eye movement; WASO—wake after sleep onset; CPAP—continuous positive airway pressure therapy.

**Table 3:** Changes in Respiratory, Cognitive, and P300 ERP Parameters Before and After 3 Months of CPAP Therapy.

	Median (IQR)		^†^ Difference	95% CI	*p* * Value
	Before CPAP	After CPAP			
AHI (events/h)	56.2 (41.1–71.7)	3.0 (2.0–4.9)	−53.4	−58.3 to −48.7	**<0.001**
ESS (daytime sleepiness)	11 (7.3–15.0)	2.0 (1.0–3.0)	−9	−10 to −8	**<0.001**
Total MoCA score	22 (19–25)	26 (25–28)	4	3 to 5	**<0.001**
P300–Event-related potentials					
P1 latency (ms)	38 (26–48)	31 (25–41)	−5	−8 to −1	**0.02**
N1 latency (ms)	95 (90–108)	95 (85–104)	−1	−5 to 3	0.50
P2 latency (ms)	184 (171–198)	176 (165–194)	−3	−8 to 3	0.39
N2 latency (ms)	238 (225–265)	236 (222–260)	−6	−13 to 0.5	0.08
P300 latency (ms)	341 (325–352)	313 (302–324)	−23	−29 to −19	**<0.001**
P300 amplitude (µV)	9.7 (6.1–13.9)	10.8 (7.4–14.5)	0.7	−0.05 to 1.6	0.07

* Wilcoxon signed-rank test; ^†^ Hodges–Lehmann median difference. Bold value denotes statistical significance. Abbreviations: IQR—Interquartile Range; AHI—apnea–hypopnea index (events per hour); ESS—Epworth Sleepiness Scale; MoCA—Montreal Cognitive Assessment; CPAP—continuous positive airway pressure therapy. Bold values denote statistical significance.

**Table 4:** Differences Between Patients With and Without HTN After 3 Months of CPAP Therapy.

	Median (IQR)		^†^ Difference	95% CI	*p* * Value
	Without HTN (n = 30)	With HTN (n = 41)			
AHI (events/h)	2.9 (2.1–4.0)	3.0 (2.0–5.2)	0.2	−0.6 to 1.2	0.59
ESS score	2 (1–3)	1 (1–3)	0	−1 to 0	0.44
Total MoCA	27 (25–28)	26 (24–28)	0	−1 to 1	0.54
P300–Event-related potentials					
P1 latency (ms)	31 (24–44)	35 (25–40)	−1	−6 to 4	0.71
N1 latency (ms)	94 (86–107)	96 (85–101)	−1	−8 to 6	0.74
P2 latency (ms)	177.5 (165–191)	175 (164.8–195)	0	−10 to 10	0.99
N2 latency (ms)	225 (219–249)	238 (226–265)	9	−5 to 21	0.21
P300 latency (ms)	307 (298–324)	319 (309–328)	9	0 to 17	0.05
P300 amplitude (µV)	9.5 (7.2–14.7)	11.1 (7.5–13.3)	0.5	−1.8 to 2.7	0.67

* Mann–Whitney U test; ^†^ Hodges-Lehmann median difference. Abbreviations: IQR—Interquartile Range; HTN-HTN; AHI—apnea–hypopnea index (events per hour); ESS—Epworth Sleepiness Scale; MoCA—Montreal Cognitive Assessment; CPAP—continuous positive airway pressure therapy. Bold values denote statistical significance.

**Table 5:** Cognitive status based on MoCA score before and after CPAP therapy by HTN status.

	Number (%) of Participants			*p* * Value
	Without HTN (n = 30)	With HTN (n = 41)	Total (n = 71)	
MoCa before CPAP				
Normal cognitive function (MoCA ≥ 26)	7 (23)	6 (15)	13 (18)	0.37
Impaired cognitive function (MoCA < 26)	23 (77)	24 (85)	58 (82)	
MoCa after CPAP				
Normal cognitive function (MoCA ≥ 26)	10 (33)	15 (37)	25 (35)	0.81
Impaired cognitive function (MoCA < 26)	20 (67)	26 (63)	46 (65)	

* Fisher’s Exact test. Abbreviations: HTN—Arterial hypertension; MoCA—Montreal Cognitive Assessment; CPAP—continuous positive airway pressure therapy.

**Table 6:** Prediction of * poor cognitive outcome at follow-up (Multivariate Logistic Regression–Stepwise Method).

	β	Wald	*p* Value	OR (95% CI)
Without adjustment for HTN				
Mean oxygen saturation	–0.13	4.23	0.04	0.87 (0.78–0.98)
Percentage of CPAP use	0.08	5.71	0.02	1.09 (1.02–1.16)
Constant	3.55	0.37	0.54	—
Adjusted for HTN				
Mean oxygen saturation	–0.13	4.11	0.04	0.88 (0.77–0.98)
Percentage of CPAP use	0.09	5.84	0.02	1.10 (1.02–1.17)
Constant	3.07	0.27	0.60	—

* Poor cognitive outcome at follow-up was coded as 1, whereas preserved cognitive performance was coded as 0. Abbreviations: Arterial hypertension–HTN; CPAP—continuous positive airway pressure therapy.

**Table 7:** ROC Analysis for Discriminating Poor Cognitive Outcome at Follow-Up.

Factor	AUC	95% CI	Sensitivity (%)	Specificity (%)	Cut-Off	Youden Index	*p* Value
Mean oxygen saturation (%)	0.630	0.507–0.742	28.0	93.5	≤85	0.215	0.06
CPAP usage (%)	0.645	0.522–0.755	96.0	30.4	>79	0.264	0.03

Abbreviations: CPAP—continuous positive airway pressure therapy.

## Data Availability

The original contributions presented in this study are included in the article. Further inquiries can be directed to the corresponding author.

## Abbreviations

The following abbreviations are used in this manuscript:

AHI	Apnea–hypopnea index
HTN	Arterial hypertension
BAER	Brainstem auditory evoked response
CPAP	Continuous positive airway pressure
ERP	Event-related potentials
OSA	Obstructive sleep apnea
